# Ocean acidification causes mortality in the medusa stage of the cubozoan *Carybdea xaymacana*

**DOI:** 10.1038/s41598-019-42121-0

**Published:** 2019-04-04

**Authors:** Pierre J. C. Chuard, Maggie D. Johnson, Frédéric Guichard

**Affiliations:** 1 Department of Biological Sciences, Bishop’s University, Sherbrooke, QC J1M 1Z7 Canada; 2 Smithsonian Marine Station, Fort Pierce, FL USA; 3 Department of Biology, McGill University, Montreal, QC H3A 1B1 Canada

## Abstract

Ocean pH is decreasing due to anthropogenic activities, and the consequences of this acidification on marine fauna and ecosystems are the subject of an increasing number of studies. Yet, the impact of ocean acidification (OA) on several abundant and ecologically important taxa, such as medusozoans, is poorly documented. To date there have been no studies on the effect of post-2050 OA projections on the medusa stage of jellyfish. As medusae represent the reproductive stage of cnidarians, negative impacts on adult jellyfish could severely impact the long-term survival of this group. Using a laboratory experiment, we investigated the effect of 2300 OA projections (i.e. pH of 7.5) on the mortality rate of the medusa-stage of the cubozoan species *Carybdea xaymacana*, compared to ambient seawater pH conditions (i.e. pH of 8.1). After a 12-h exposure to OA, *C*. *xaymacana* medusae suffered higher mortality rates compared to ambient conditions. This study represents the first evidence of the potential lethal effects of post-2050 OA projections on jellyfish. The higher metabolic rates of cubozoans compared to other cnidarians might make box jellyfish more vulnerable to OA. A decrease in the density of cnidarians could lead to harmful ecological events, such as algal blooms.

## Introduction

By 2300, surface ocean pH levels are predicted to decrease by 0.67 units compared to pre-industrial levels^1^. These altered pH conditions (known as ocean acidification; OA) are a result of increasing anthropogenic carbon dioxide emissions^2^. It is well established that OA has detrimental effects on biological processes in several marine organisms, particularly calcification of invertebrates^3^. Other potential harmful effects of OA include acidosis^4^, a process that can lead to reduced metabolic rates^5^.

Jellyfish populations seem to have grown recently (but see Condon *et al*.^6^) and have the potential to dominate surface water biomass^7^ (i.e. jelly blooms). The increasing biomass of jellyfishes, sometimes followed by waves of high mortality, is suspected to influence the ocean biological pump^8^. Indeed, these high-mortality events allow for more carbon to be sequestered into the ocean floor, and thus increase the capacity of oceans to act as CO_2_ sinks. The ocean biological pump is a key mechanism transferring nutrients from surface to deep-ocean ecosystems^9^, thus connecting ocean food webs over large scales. However, we know little about the biology of many regionally dominant jellyfish species^10^. Moreover, though specific-mechanisms causing jellyfish blooms are uncertain^7,11^, climate-driven environmental changes likely play an important role. Understanding the effects of climate-driven stressors on jellyfish is essential to determining their role within changing ocean ecosystems^10,12^ (e.g. changes in contribution to climate change mitigation through increase or decrease in population size).

Research on the effects of changes in pH on jellyfish species remains limited (Table 1). Within possible long-term OA projection scenarios (i.e. using alkaline pH treatments), these few studies found positive and negative non-lethal effects of pH on jellyfishes, depending on life stages and species, and have led to the general perception that jellyfish are relatively resilient to OA^13^. Thus far, only a pH below 4.5 (far more acidic than OA projections) led to mortality in polyps of the moon jellyfish *Aurelia aurita*^14^. Only one study investigated the reproductive life stage of jellyfish (i.e. medusa)^13^ in response to short-term (2050) OA projections, and no study has investigated the effects of longer-term OA scenarios on medusae despite their importance for the maintenance of genetic diversity within populations. More empirical evidence is necessary to determine the overall resilience of jellyfishes to OA using more species and understudied life-stages.

**Table 1:** Details from all known studies that investigated the effects of pH on jellyfish species.

Study	Class	Species	Life stages	Lowest pH treatment	Effects
Kikkawa *et al*.^29^	Scyphozoa	*Aurelia sp*.	Ephyra	6.15	Swimming inhibited (pH ≤ 6.37) and inverted arms
Winans and Purcell^43^	Scyphozoa	*Aurelia labiata*	Polyp; ephyra	7.2	Smaller statoliths in ephyrae
Klein *et al*.^23^	Cubozoa	*Alatina* nr *mordens*	Polyp	7.6	Slower reproduction
Lesniowski *et al*.^44^	Scyphozoa	*Cyanea capillata*; *Chrysaora hysoscella*	Polyp	7.9	None
Alguero-Muniz *et al*.^45^	Scyphozoa	*Aurelia aurita*	Ephyra	7.28	Slower growth
Tills *et al*.^46^	Scyphozoa	*Aurelia aurita*	Ephyra	7.6	Smaller size and lower pulsation rate
Goldstein *et al*.^14^	Scyphozoa	*Aurelia aurita*	Planula larva; polyp	7.4 (larva) 2 (polyp)	Increased larvae settlement and tissue degradation (pH < 6.5) and mortality (pH < 4.5) of polyps
Klein *et al*.^24^	Cubozoa	*Alatina alata*	Polyp	7.55	Reproductive and protein content resilience, and increased early respiration rate under predicted temperature increase. Decreased prey capture rates
Hammill *et al*.^13^	Cubozoa	*Carybdea rastoni*	Medusa	7.77	Increased foraging rates
Treible *et al*.^47^	Scyphozoa	*Aurelia aurita*	Polyp	7.62	None

*Carybdea xaymacana* Conant, 1897 is an abundant cubozoan found in most coastal waters in the tropics and subtropics^15^. *C*. *xaymacana* is a small box jellyfish (i.e. bell height <3 cm) with four arms each connecting a tentacle to the box-shaped bell that is characteristic to cubozoans^16–18^. With tentacles expanding up to 20 cm, it feeds on zooplankton and small pelagic organisms^18^. *C*. *xaymacana* inhabits calm bays, including our study site in the Caribbean Sea (Bahía de Almirante, Panama). To our knowledge, *C*. *xaymacana* has not been the focus of experimental manipulations other than basic taxonomic descriptions^18^, and some studies investigating the venomousness for which cubozoans are infamous^19,20^. The Cubozoa class (Cnidaria) encompasses approximately 50 known species^21^, whereas the Scyphozoa class (Cnidaria) includes more than 200 described species^22^. This difference, along with the fact that scyphozoans are usually the cause of jelly blooms^7^, might explain the lower representation of cubozoans compared to scyphozoans in the OA literature (i.e. 3 species; Table 1). The effects of OA on box-jellyfishes has only been investigated in three other species (*Alatina* nr *mordens*^23^, *Alatina alata*^24^, and *Carybdea rastoni*)^13^ down to a pH of minimum 7.55^24^, which does not encompass 2300 OA projections (i.e. 7.5^1^).

In the present study, we address these knowledge gaps by investigating long-term OA scenario (pH = 7.5 in 2300^1^) effects on the medusa life stage of *C*. *xaymacana*. We exposed groups of individuals in the laboratory to either ambient (pH = 8.1) or reduced pH (pH = 7.5) seawater. We predicted that jellyfish would survive our lower experimental pH treatment given the reported resilience of jellyfish to OA (Table 1). Given the physiological differences between scyphozoan and cubozoan medusae (e.g. higher metabolic rates in box jellyfishes compared to true jellyfishes)^25^, studies investigating the effects of post-2050 OA projections on adult cubozoans are needed to improve predictions regarding the fate of cnidarians in changing oceans.

## Results

### Treatment conditions

As expected, optical dissolved oxygen (ODO) and salinity were similar between treatments. We were able to maintain treatment conditions throughout the duration of the experiment (Table 2). Indeed, ODO and salinity were 5.93–6.10 mg l^−1^, and 35 psu for the control; 5.88–6.12 mg l^−1^, and 36 psu for the reduced pH treatment respectively. These values were comparable to the sampling site (7.42 mg l^−1^, 36 psu for ODO and salinity respectively). The average of the two measurements of pH_T_ and A_T_ in the reduced pH treatment were 7.52 and 2282 µmol kg^−1^ of seawater respectively. The differences between the two measurements for both pH_T_ and A_T_ were small (<1% difference between measurements on average). The single measurement of the control treatment between the two trial blocks had a pH_T_ of 8.10, comparable to the seawater pH_T_ measured at the sampling site a few minutes before (8.07 pH_T_). Finally, pCO_2_ of the control treatment was 511 µatm.

**Table 2:** Physical parameters for each treatment.

Treatment	T (°C)	Salinity (psu)	pH^‡^	A_T_ (*μ*mol kg^−1^)	pCO_2_ (µatm)	DIC (*μ*mol kg^−1^)	Ω_Αr_
Control	28 (±0.4)	2279	8.1	2279	511	2003	3.12
Reduced pH	28 (±0.4)	2277–2287	7.48–7.55	2277–2287	2115–2579	2236–2273	0.89–1.05

We measured temperature twice daily. We measured salinity, and collected water sampled for total alkalinity (A_T_) and pH once for the control treatment (i.e. between the two trial blocks), and twice for the reduced pH treatment (i.e. between and after the two trial blocks). We derived pCO_2_, DIC, and Ω_Ar_ from measured values of A_T_, salinity and pH using CO2SYS. ^‡^NBS scale.

### Mortality rates

We observed mortality at a rate of 35% (±19% s.e.m.) in the reduced pH treatment after 12 h and no mortality in the control treatment. These mortality rates were significantly greater in the reduced pH treatment than in the control (regression coefficient: 3.79, [95% confidence interval: 0.89, 6.69], *z* score: 2.57, *P* = 0.010). In addition, compared to the control, most living individuals in the reduced pH treatment displayed signs of poor health, such as general lethargy, loss of tentacles, everted bells, and ineffective bell movement. We also observed retractions of tentacles inside the bell in some individuals in the reduced pH treatment, without apparent prey capture. In contrasts, *C*. *xaymacana* individuals from the control treatment were freely swimming at the end of trials.

## Discussion

Our results show increased in mortality of *C*. *xaymacana* under 2300 ocean acidification projections (i.e. pH of 7.5^1^). This study represents the first evidence of lethal effects of future OA conditions on a jellyfish species (Table 1). To our knowledge, no known medusa stage or cubozoan species of any life stage has been experimentally exposed to a pH of 7.5 or lower before. As the effect of OA seems to be taxon-specific^26^, cubozoans might be more sensitive to OA than other studied jellyfish species. A severe increase in mortality of jellyfish reproductive stage could impair their potential to adapt to climate change by reducing the genetic diversity of their populations^27^. Other marine invertebrates have shown similar sensitivity to changes in ocean pH, the most vulnerable ones being squids^28^. Their high metabolic rates, among other factors, make squids more vulnerable to OA than other invertebrates.

A decrease in water pH has been shown to create acidosis in some organisms, potentially causing a reduction in metabolic rates^5^. Such reduction can lead to the shutdown of non-vital but energetically costly metabolic processes^4^. For example, the swimming abilities of the ephyrae of *Aurelia* sps. are totally inhibited at a pH of 6.37^29^. In nature, this inaptitude would likely result in an increase in susceptibility to predation and starvation. Given the unhealthy state in which most of the remaining living individuals were found in the reduced pH treatment after the experiment (e.g. tentacle retraction, which is a characteristic distress behaviour in sea anemones^30^, another cnidarian), it appears that vital functions of medusae were also affected by a reduction in pH. Similar to squids, the high metabolic activity of cubozoans relative to other jellyfish species (e.g. scyphozoans)^25,31^ may heighten box jellyfish sensitivity to changes in their environment (e.g. pH)^28,29^. This hypothesis is supported by the rarity of *C*. *xaymacana* inside mangrove ecosystems in Bahía de Almirante despite the presence of appropriate prey^32^. Indeed, mangrove pools in the region seem to undergo diurnal shifts in water pH conditions, dropping as low as 6.77 pH during the night^32^. In comparison, pH at our sampling site was higher than in the mangrove pools^32^. Although cubozoans might be more vulnerable to laboratory conditions than other jellyfish species (Jimena Garcia-Rodriguez personal communication), they have been cultivated successfully in the laboratory over periods of days^13^ to generations^23,24^ within the context of OA experiments.

Independent of the proximate causes behind the observed increase in mortality of *C*. *xaymacana* under OA, our study suggests that negative effects of OA in the future lead to high mortality in jellyfish and a decrease in the number of individuals/populations of this species, especially in the medusa stage. The adult life stage of jellyfish (i.e. medusa) is responsible for maintaining genetic diversity among and within populations through sexual reproduction and high mobility^33^. A strong decrease in the population size of jellyfishes, especially of their reproductive stage, would reduce genotypic variation^34^, which might limit their adaptability and resilience in the face of environmental changes^35^. As *C*. *xaymacana* is abundant in various parts of the world and is a generalist predator of zooplankton^18^, a decrease in the abundance of *C*. *xaymacana* could have important indirect impacts on marine ecosystems. An increase in the mortality of this species would decrease predation pressures on zooplankton communities. Due to the high abundance of zooplankton in the ocean, even slight decreases in their mortality can destabilize whole ecosystems^36^. For example, an increase in mesoplankton (e.g. due a decrease in the number of medusae) would lead to a top-down effect on lower-level plankton communities^37^. An increase in phytoplankton biomass could increase the occurrence of algal blooms^38^ that can harm ecosystems due to their toxicity. Other cross-ecosystem effects of an increase in jellyfish mortality could involve the ocean biological pump^8^, and affect deep-sea ecosystems without cubozoans.

Our study provides evidence for potential lethal effects of OA on the medusa stage of cubozoans. This finding questions the prevailing theory that jellyfish are resilient to climate change, and provides evidence that some members of this group suffer mortality. Future research should replicate similar experiments with other species at the medusa stage under various pH conditions to see if these findings are generalizable. In addition, we recommend the investigation of the proximate causes of the mortality in *C*. *xaymacana* under OA (e.g. chemical component analysis of tissue), along with the impact of jellyfish on ocean ecosystems in a rapidly changing environment.

## Materials and Methods

### Collection of individuals

We conducted this experiment over 2 days in October 2017, at the Smithsonian Tropical Research Institute’s (STRI) Bocas del Toro Research Station on the Caribbean coast of Panama. We collected individuals near the dock of the research station. As *C*. *xaymacana* forages close to the water surface at night and is attracted to light^18^, we caught individuals at nightfall using a flashlight. We used a 2-L bucket to capture jellyfish along with some seawater. We then brought jellyfish back to the wet laboratory facilities at the station.

### Experimental design

We assigned jellyfish individuals haphazardly to one of four 40-L test-buckets, using 10 individuals per replicate bucket. We assigned half the buckets to the control treatment (i.e. known to fluctuate daily between a total scale pH (pH_T_) of 7.92 and 8.05)^39^ and half of them to the reduced pH treatment (i.e. 7.5 pH).

We brought each test-bucket to their treatment conditions before adding individuals. Abrupt pH changes are common when individuals move through space in their natural environment^32^ (e.g. up to 1.51 difference in pH units). Ambient seawater was pumped from a depth of ∼3 meters adjacent to the Smithsonian Tropical Research Institute’s (STRI) dock and was passed through a 50-µm filter (Bubble Bead, Aquaculture Systems Technologies). We plumbed ambient seawater into control buckets or a 120-L reservoir tank (header tank) for pH manipulations (i.e. pH of 7.5; see Treatment conditions). The acidified water was then pumped into the test-buckets assigned to the reduced pH treatment through airline tubing at a rate of 15 ml min^−1^. For the control test-buckets, ambient seawater from the station seawater system was plumbed into control buckets at the same rate. Excess water flowed over the top of the test-buckets, and because the flow rate was slow we did not observe any loss of jellyfish. We did not feed individuals in the laboratory. We maintained a constant temperature of 28 °C (±0.4) using aquarium heaters in all test-buckets. We checked the temperature twice a day in each test-bucket throughout the experiment, before and after each trial block. We also equipped each test-bucket with a small submersible water pump to create a slow current and keep jellyfish from settling to the bottom. We placed each piece of equipment so that they were far enough from the walls of test-buckets to avoid jellyfish congestion.

Each trial ran for 12 h on a 12:12 hour light cycle. We chose a 12-h period as pilot experiments showed mortality in that timeframe, and because this time of exposure to low pH conditions is longer than what this population is naturally exposed to (i.e. less than 6 h)^32^. We ran two blocks of four trials each (i.e. 2 control trials and 2 reduced pH trials per block) for a total of 80 tested individuals. At the end of each trial block, we haphazardly selected a test bucket within each treatment to measure ODO and salinity using a multiparameter probe (YSI, Exo). In addition, we measured those parameters at the sampling site once between the two trial blocks as reference.

### Treatment conditions

To expose *C*. *xaymacana* to 2300 pH projections (i.e. pH of 7.5^1^), we bubbled ambient seawater in a header tank with pure CO_2_ using a pH-feedback system (Neptune Apex). The header tank was made from a 75-L industrial garbage can with constant flow of ambient seawater that we mediated by an automated float valve. We placed 2 submersible aquarium pumps (1600 l h^−1^) in the header tank. One pumped treatment seawater from the header tank, through a manifold, and into treatment buckets. We connected an airline to a venturi injector on the second pump to facilitate circulation and rapid diffusion of pure CO_2_ gas within the header tank. pH of header tanks was monitored continuously with a pH probe (Neptune) that was connected to a solenoid valve through an Apex control unit. Target pH values were set to 7.5. The solenoid valve triggered on, releasing pure CO_2_ into the header tank, when pH exceeded the target value, and off when pH returned to the set range. We optimized the flow rate of CO_2_ into the header tank to minimize deviation from target pH to within 0.1, which represents a maximum fluctuation of 0.03 units of pH during trials in the reduced pH treatment due to the low pumping rate. Johnson *et al*.^39^ measured total scale pH_T_ daily in their ambient seawater treatments at our laboratory facility, providing evidence that pH deviations in our control trials were likely to be of a similar range (i.e. maximum 0.07 units of pH_T_) compared to reduced pH treatments. We calibrated the Neptune pH probe with NBS buffers following factory protocol (pH 7.00, 10.00).

We collected water samples between the two trial blocks and from the reduced pH treatment after the end of the experiment for measurements of pH and total alkalinity. We collected the samples outside of test buckets (i.e. control sample from the distributed seawater in the laboratory and reduced pH sample from the header tank) and treated samples with 200 µL of saturated HgCl_2_ solution to prevent biological alteration of carbonate parameters. We determined pH_T_ and total alkalinity (A_T_) for each sample. We measured pH_T_ (total scale) and total alkalinity (A_T_) using an automated titrator (Mettler Toledo DL15) and modified open-cell potentiometric titrations. The titrator was fitted with a glass electrode (Mettler Toledo, DG115-SC) that was calibrated at the start of titrations with NBS buffers (pH 4.00, 7.00, 10.00). pH was determined at the start of titrations. We checked the quality of A_T_ measurements for accuracy against certified reference material (Reference Material for Oceanic CO_2_ measurements, Batch 158, A. Dickson) at the start and end of titrations. The mean accuracy of A_T_ measurements was 0.44% (N = 4). We calculated the remaining carbonate parameters (i.e. pCO2, dissolved organic carbon (DIC), and aragonite saturation state (Ω_Ar_)) from measured values of A_T_, salinity and pH using CO2SYS. All output tank calculations were based on tank temperatures of 28 °C.

### Mortality rates

We measured mortality 12 h after initiation of a trial. We considered an individual to be alive if it was pulsing, including after being stimulated by a pipette. After a trial block, we released surviving individuals on the opposite end of the dock in more open water compared to the collection site to avoid resampling. Between the two trial blocks, we emptied and cleaned test-buckets, and let them refill with the corresponding treatment water as described above.

### Statistical analysis

We used a two-tailed generalized linear model (GLM) fitted to a binomial distribution, and linked to a logit function, to test for the effect of pH treatment on mortality rates. A data point consisted of the 10 individuals from a single trial (i.e. number of individuals alive vs. dead). We included pH treatment as a fixed factor. We performed all statistical tests in R^40^ using the *brglm* package^41^ which accounts for complete separation of the data^42^ (i.e. mortality observed in the reduced pH treatment only) with an α level of 0.05.

### Ethical Statement

All work reported herein was approved by the STRI Animal Care and Use Committee (protocol #2017-0905-2020).

## Data Availability

We made the data publicly accessible on FigShare for both the dataset (https://figshare.com/s/9e98195503d34eb46e73) and the analysis script (https://figshare.com/s/b2e0ab822e54da8138b4).
